# Trends in the incidence of head and neck cancer by subsite between 1993 and 2015 in Japan

**DOI:** 10.1002/cam4.4539

**Published:** 2022-01-14

**Authors:** Daisuke Kawakita, Isao Oze, Shinichi Iwasaki, Tomohiro Matsuda, Keitaro Matsuo, Hidemi Ito

**Affiliations:** ^1^ Division of Cancer Epidemiology and Prevention Department of Preventive Medicine Aichi Cancer Center Research Institute Nagoya Japan; ^2^ Department of Otorhinolaryngology, Head and Neck Surgery Nagoya City University Graduate School of Medical Sciences Nagoya Japan; ^3^ Division of International Collaborative Research Center for Public Health Sciences National Cancer Center Tokyo Japan; ^4^ Department of Epidemiology Nagoya University Graduate School of Medicine Nagoya Japan; ^5^ Division of Cancer Information and Control Department of Preventive Medicine Aichi Cancer Center Research Institute Nagoya Japan; ^6^ Division of Descriptive Cancer Epidemiology Nagoya University Graduate School of Medicine Nagoya Japan

**Keywords:** age‐standardized incidence rate, annual percent change, head and neck cancer, subsite

## Abstract

**Background:**

Tobacco use and alcohol consumption are still important risk factors for head and neck cancer (HNC) in developing countries, even though decreasing in tobacco prevalence. Recently, an increased incidence of oropharyngeal cancer due to human papilloma virus (HPV) infection has attracted attention in advanced countries, including the United States and Europe. However, few studies have evaluated trends in the incidence of HNC by subsite in Japan.

**Methods:**

Accordingly, we evaluated these trends in Japan using data from population‐based cancer registries. We compiled population‐based incidence data from the Monitoring of Cancer Incidence in Japan Project, based on data from 19 population‐based cancer registries. Number of incident cases and age‐standardized incidence rates of HNC were estimated by subsite, namely lip, oral cavity, salivary glands, nasopharynx, oropharynx, hypopharynx, larynx, nasal and paranasal cavity, middle ear and NOS. Trends in agestandardized incidence rates were characterized using the Joinpoint analysis.

**Results:**

Among both sexes, oral cavity cancer, salivary gland cancer, and oropharyngeal cancer showed an upward trend (oral cavity: annual percent change (APC) 1.2% for men and APC 1.9% for women; salivary gland: APC 2.2% for men and APC 3.1% for women; oropharynx: APC 5.0% for men and APC 7.6% for women). Additionally, hypopharyngeal cancer showed an upward trend for men (APC 4.1%), and nasopharyngeal cancer and laryngeal cancer showed a downward trend for men (nasopharynx: APC −2.7%; larynx: −1.1%).

**Conclusions:**

These findings will assist in focusing on the individual prevention of HNC.

## Graphical abstract

This article describe trends in the incidence of head and neck cancer by subsite between 1993 and 2015 in Japan. These findings could assist in focusing on the individual prevention of head and neck cancer in Japan.

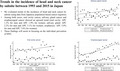

## 1 INTRODUCTION

Head and neck cancer (HNC) is the seventh most common cancer worldwide, and accounts for over 800,000 new cases annually.^1^ HNC occurs in various subsites, including the lip, oral cavity, salivary glands, nasopharynx, oropharynx, hypopharynx, larynx, nasal and paranasal cavity, and ear. Indeed, the most common sites of incidence among HNC cases vary by geographic region, because the etiology of HNC is mainly lifestyle factors.^2^, ^3^, ^4^, ^5^, ^6^ Tobacco use and alcohol consumption are still important risk factors for HNC in developing countries, even though decreasing in tobacco prevalence.^7^ In contrast, the incidence of oropharyngeal cancer due to human papilloma virus (HPV) infection has been increasing in advanced countries, including United States and European countries.^8^, ^9^, ^10^ Thus, an understanding of trends in the incidence of HNC by subsite is one of the most important aspects of management of this condition. To date, however, few studies have evaluated trends in the incidence of HNC by subsite in Japan.^11^, ^12^, ^13^

Here, we assessed the overall incidence of HNC among Japanese men and women between 1993 and 2015, and describe the distribution and trends in incidence rates of HNC at subsites.

## 2 MATERIALS AND METHODS

### 2.1 Populations

We used data from 19 population‐based cancer registries in Kumamoto, Nagasaki, Saga, Yamaguchi, Hiroshima, Tottori, Osaka, Shiga, Aichi, Fukui, Niigata, Kanagawa, Chiba, Gunma, Tochigi, Ibaragi, Yamagata, Miyagi, and Aomori prefectural governments, all of which are members of the Monitoring of Cancer Incidence in Japan (MCIJ) project.^14^ All 47 prefectural cancer registries in Japan submitted cancer incidence data to the Project. National statistics of cancer incidence in 2015 in Japan were observed using date submitted to the MCIJ project in 2015. First, we selected 41 prefectural cancer registries whose data quality for all cancers in 2015 met the following the standards in the MCIJ project 2015: (i) proportion of cases reported by death certificate only (DCO%: death certificate only) of less than 10%, (ii) proportion of cases first notified through death certificate (DCN%: death certificate notification) of less than 20%, (iii) and mortality to incidence ratio (M/I) of less than or equal to 0.5.^14^ From these, we then selected the 19 registries above because they submitted data between 1993 and 2005 to the MCIJ project 2015.^14^ The selected registries encompassed data for 44.6% of the total Japanese population in 2015.

### 2.2 Disease coding

Topology codes of the International Classification of Diseases, Version 10 (ICD10) were grouped into 10 categories: lip (C00), oral cavity (C02‐04, 05.0, 05.8, 05.9, 06), salivary glands (C07‐08), nasopharynx (C11), oropharynx (C01, 05.1, 05.2, 09–10), hypopharynx (C12‐13), larynx (C32), nasal and paranasal cavity (C30.0 and C31), middle ear (C30.1), and oral cavity or pharynx not otherwise specified (NOS) (C14). Epithelial malignancies (8000–8574, ICD‐O‐3) arising in the head and neck were included.

### 2.3 Statistical methods

We estimated sex‐specific incidence rates of each subsite per 100,000 person‐years and 95% confidence intervals (CI). Each incidence rate was standardized by age‐adjustment according to the Segi's world standard population.^15^ In addition, the annual percent change (APC) was calculated using the Joinpoint regression analysis.^16^, ^17^ Briefly, the Joinpoint regression analysis is a statistical method for the analysis of change in trends over continuous segments of time. The significance of an increase or decrease within each segment was evaluated after identifying the best fitting model. In describing the trends, if changing less than or equal to 0.5% per year (−0.5 ≤ APC ≤ 0.5) and the APC was not statistically significant, we characterized it as stable. If changing more than 0.5% per year (APC < −0.5 or APC > 0.5) and the APC was not statistically significant, we characterized it as non‐significant change. If changing with a statistically significant APC > 0, we characterized it as rising. If changing with a statistically significant APC < 0, we characterized it as falling. We estimated age‐adjusted incidence rates with STATA version 16 (STATA Corporation). For the Joinpoint regression analysis, we used the Joinpoint Regression Program version 4.9.0.0 (US National Cancer Institute). For the Joinpoint regression analysis, we considered that less than 0.05 of two‐sided *p*‐values were statistically significant.

## 3 RESULTS

Table 1 shows estimated age‐standardized incidence rates (ASRs) per 100,000 men and women for HNC according to subsite in 2015. The ASR of overall HNC was estimated as 12.42 per 100,000 men and 3.71 per 100,000 women. The leading subsite was oral cavity among both men and women (ASR: 3.40 per 100,000 men and 1.93 per 100,000 women). Among men, larynx was the second most common site, followed by hypopharynx (ASR: 2.82 per 100,000 men for larynx; 1.93 per 100,000 men for hypopharynx). Additionally, salivary gland was the second most common site among women, followed by oropharynx (ASR: 0.44 per 100,000 women for salivary gland; 0.41 per 100,000 women for oropharynx).

**TABLE 1:** Age‐standardized incidence rates per 100,000 men and women for head and neck cancer by subsite in 2015

Subsite	ICD10 code	Men		Women	
		ASR	%	ASR	%
Overall		12.42		3.71	
Lip	C00	0.05	0.4	0.03	0.8
Oral cavity	C02‐04, 05.0, 05.8, 05.9, 06	3.40	27.4	1.93	52.0
Salivary gland	C07, 08	0.68	5.5	0.44	11.8
Nasopharynx	C11	0.49	4.0	0.16	4.2
Oropharynx	C01, 05.1, 05.2, 09, 10	1.93	15.5	0.41	11.0
Hypopharynx	C12, 13	2.25	18.1	0.23	6.1
Larynx	C32	2.82	22.7	0.25	6.7
Nasal and paranasal cavity	C30.0, 31	0.71	5.7	0.25	6.7
Middle ear	C30.1	0.01	0.1	0.01	0.2
NOS	C14	0.07	0.6	0.02	0.5

Abbreviations: ASR, age‐standardized incidence rate; ICD, International Classification of Diseases; NOS, oral cavity or pharynx not otherwise specified.

**FIGURE 1:**
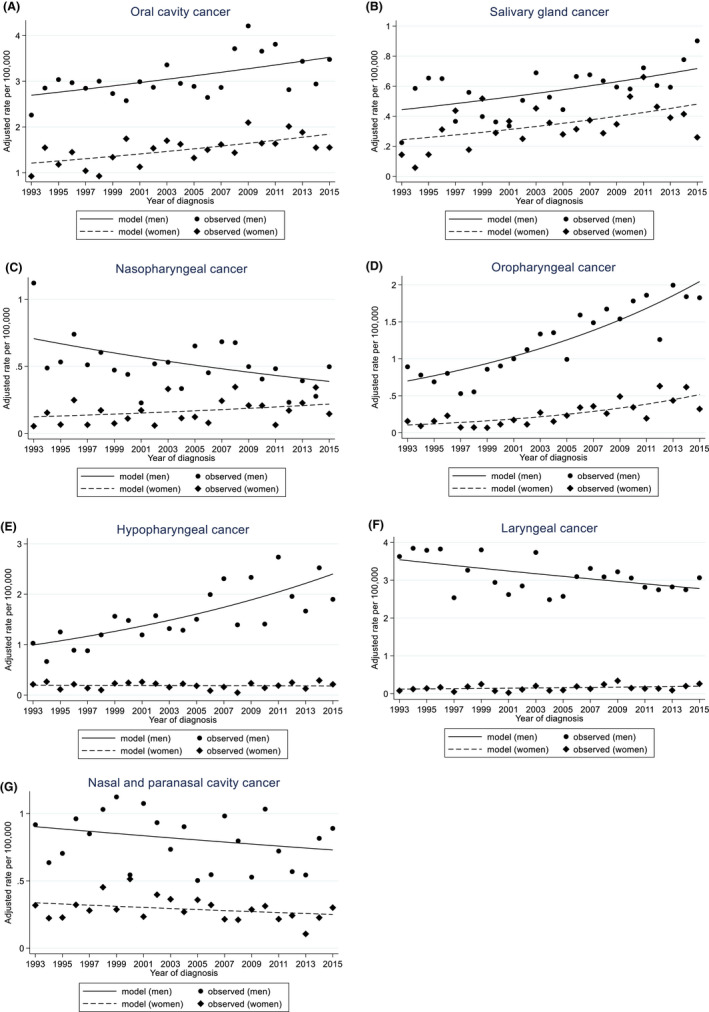
Joinpoint analysis for age‐standardized (world population) incidence rates of oral cavity (A), salivary gland (B), nasopharyngeal (C), oropharyngeal (D), hypopharyngeal (E), laryngeal (F), nasal and paranasal cavity cancer (G) per 100,000 men and women

Figure 1, Table 2, Table S1, and Figure S1 showed the results of the Joinpoint regression analyses for HNC incidence trends according to subsite between 1993 and 2015. All HNC showed an upward trend between 1993 and 2015 among both men and women (APC 0.9%, 95% CI: 0.3% to 1.5%, for men; APC 2.1%, 95% CI: 1.2% to 3.0%, for women). Oral cavity cancer, salivary gland cancer, and oropharyngeal cancer showed an increasing trend during the study period among both men and women (oral cavity: APC 1.2%, 95% CI: 0.4% to 2.1%, for men and APC 1.9%, 95% CI: 0.8% to 3.1%, for women; salivary gland: APC 2.2%, 95% CI: 0.6% to 3.9%, for men and APC 3.1%, 95% CI: 0.5% to 5.8%%, for women; oropharynx: APC 5.0%, 95% CI: 3.8% to 6.2%, for men and APC 7.6%, 95% CI: 4.7% to 10.5%, for women). Among men, hypopharyngeal cancer showed an increasing trend (APC 4.1%, 95% CI: 2.5% to 5.7%). In addition, nasopharyngeal cancer and laryngeal cancer showed a downward trend for men (nasopharynx: APC −2.7%, 95% CI: −4.6% to −0.7%; larynx: APC −1.1%, 95% CI: −1.9% to −0.3%). We did not find a significant trend for nasal and paranasal cavity cancer.

**TABLE 2:** Joinpoint analysis for head and neck cancer by subsite between 1993 and 2015

Subsite	Year	Men		Year	Women	
		APC	95% CI		APC	95% CI
Overall	1993–2015	0.9*	0.3, 1.5	1993–2015	2.1*	1.2, 3.0
Oral cavity	1993–2015	1.2*	0.4, 2.1	1993–2015	1.9*	0.8, 3.1
Salivary glands	1993–2015	2.2*	0.6, 3.9	1993–2015	3.1*	0.5, 5.8
Nasopharynx	1993–2015	−2.7*	−4.6, −0.7	1993–2015	2.6	−0.9, 6.3
Oropharynx	1993–2015	5.0*	3.8, 6.2	1993–2015	7.6*	4.7, 10.5
Hypopharynx	1993–2015	4.1*	2.5, 5.7	1993–2015	−0.4	−2.9, 2.2
Larynx	1993–2015	−1.1*	−1.9, −0.3	1993–2015	2.3	−1.1, 5.7
Nasal and paranasal cavity	1993–2015	−1.0	−2.5, 0.6	1993–2015	−1.3	−3.2, 0.5

Abbreviations: APC, estimated annual percent change (age‐standardized to the world population); CI, confidence interval; NOS, oral cavity or pharynx not otherwise specified.

* APC is statistically significantly different from zero (two‐sided *p* < 0.05).

Due to the small number of incidence cases, we performed the Joinpoint regression analysis for lip, middle ear and NOS cancer using the moving average of incidence rate in 3‐year category. Middle ear cancer showed a decreasing trend for men (APC −2.2%, 95% CI; −4.4% to −0.1%, between 1994 and 2006 and APC −10.4%, 95% CI; −14.7% to −5.9%, between 2006 and 2014). We did not find a significant trend for cancer of lip and NOS.

## 4 DISCUSSION

In this study, we investigated trends in HNC incidence and site‐specific HNC incidence in a large Japanese population between 1993 and 2015. All HNC showed an increasing trend between 1993 and 2015 among both men and women. By subsite, increasing trends for men were observed for cancer of the oral cavity, salivary gland, oropharynx and hypopharynx, and for women with cancer of the oral cavity, salivary gland, and oropharynx. Additionally, decreasing trends for men were observed for cancer of the nasopharynx and larynx. To our best knowledge, this study is the first study to evaluate trends in HNC incidence according to subsite using data from Japanese population‐based cancer registries.

With regard to all HNC, a steady downward trend after 1973 was shown in the United States.^18^, ^19^ In addition, oropharyngeal cancer showed an upward trend due to the increasing the prevalence of HPV infection, and laryngeal cancer showed a downward trend, which has been attributed to decreased rates of smoking.^9^, ^18^ In England, all HNC showed an upward trend between 2002 and 2011.^20^ Additionally, HPV‐associated sites had a significantly increasing trend while laryngeal cancer had a stable trend.^20^ Consistent trends were seen in Denmark and the Netherlands.^21^, ^22^ On the other hand, in France, all HNC showed a downward trend for men and an upward trend for women between 1980 and 2012. This trend was consistent between HPV‐associated and HPV‐unassociated sites.^23^ As for the Asia, Korea showed a downward trend for all HNC in men and a stable trend in women.^24^, ^25^ In addition, the overall incidence of HNC in Taiwan has continued to increase due to a rapid rise in oropharyngeal cancer.^25^, ^26^ In Thailand, although all HNC showed a downward trend, tongue cancer for both men and women and pharyngeal cancer for men had an upward trend.^27^ In Japan, we found that all HNC had an upward trend in both men and women between 1993 and 2015. Consistent with the other countries, we found that oropharyngeal cancer had an upward trend for men and women in the study period. In addition, laryngeal cancer had a downward trend for men. Therefore, the more detailed investigation to clarify the impact of risk factors, including smoking and HPV infection, on the incidence of HNC in Japan is needed.

The increasing trend in HPV‐positive oropharyngeal cancer in the United States and European countries is well known,^8^, ^9^, ^10^, ^28^ particularly given that HPV‐positive oropharyngeal cancer cases have better survival than HPV‐negative cases.^29^ The prevalence of HPV infection in oropharyngeal cancer have been investigated in the Japanese population.^30^, ^31^, ^32^ Hama reported that the prevalence of HPV infection was 50.3% (79/157) among Japanese oropharyngeal cancer cases.^30^ In addition, Saito reported that the prevalence of p16‐positive oropharyngeal squamous cell carcinoma cases increased from 15.2% between 2000 and 2003 to 33.3% between 2008 and 2011 at a single institution.^32^ However, we were unable to find any cancer registry‐based descriptive study of trends in oropharyngeal cancer incidence in Japan. In the present study, we showed that oropharyngeal cancer has been increasing for both men and women. Gillison reported that higher prevalence of oral HPV infection among men might be associated with the higher incidence of HPV‐associated HNC.^33^ Several possible mechanisms of this association have been suggested.^26^, ^33^, ^34^, ^35^ First, regarding sexual partners, higher number of men compared to women might increase the possibility of HPV infection in oral cavity. Second, female‐to‐male transmission of HPV through oral sex is more effective compared to male‐to‐female transmission. Finally, the seroprevalence of HPV is reportedly lower in men compared to women, and high levels of antibody against HPV have been shown to protect against subsequent HPV infection.^36^, ^37^ Unfortunately, we could not directly evaluate the association with these HPV infection‐related factors because information on HPV infection was not available in this study, which is considered to be a limitation of this study. However, these factors might be associated with an upward trend in oropharyngeal cancer in Japan.

Tobacco smoking and alcohol drinking are the established risk factors for HNC.^2^, ^6^, ^38^, ^39^ Among subsites, laryngeal cancer is especially well known for its strong association with smoking.^2^, ^39^ The rate of smoking has been decreasing globally, including Japan,^2^, ^40^, ^41^, ^42^, ^43^ although in contrast to the situation for men, the prevalence of current smoking among Japanese women has remained stable.^42^, ^43^ Despite this stable trend in women—extending back to the early 2000s, and thus covering the period in which prior smoking is expected to influence cancer incidence—laryngeal cancer did not show a downward trend for women throughout the study period. This apparent discrepancy indicates the need to investigate the impact of other environmental or genetic risk factors on the incidence of laryngeal cancer among women in Japan.

Similarly, we found that hypopharyngeal cancer has increased among men. As for other countries, the Netherlands showed a downward trend for men and an upward trend for women between 1991 and 2010,^44^ while in the United States, the incidence of hypopharyngeal cancer decreased from 1973 to 2010 with an APC of −2.0% every year.^45^ Compared to other sites, hypopharyngeal cancer is more strongly associated with alcohol drinking than tobacco smoking, which is plausible considering the anatomical site.^6^, ^46^, ^47^ In fact, the prevalence of habitual alcohol drinkers showed a slightly upward trend for men until early 2000s in Japan.^42^ These trends might explain the observed increasing trends in hypopharyngeal cancer among men. It is known that hypopharyngeal cancer has the worst prognosis among HNC subtypes due to its frequent diagnosis at an already‐advanced stage.^48^ The upward trend in hypopharyngeal cancer should be noted, and focus should be placed on reducing the prevalence of the habitual alcohol drinking.

Among other findings, we found that salivary gland cancer shows an increasing trend among both men and women. Consistently with Japan, the United States showed that parotid gland cancer, which accounts for the majority of salivary gland cancers, rose steadily between 1973 and 2015 using the SEER database.^49^, ^50^ So far, several factors have been associated with the development of salivary gland cancer, including radiation or industrial exposure and smoking.^51^, ^52^, ^53^ However, due to the rarity and distinct heterogeneous histopathological subtypes, risk factors for salivary gland cancer have not been established. These results identify a clear need to clarify the risk factors for salivary gland cancer in the Japanese population.

Finally, past data quality issues in the Japanese prefectural population‐based cancer registries should be discussed. Data failed to meet international data quality standards in the early period but did do so in the later period (DCO: 26.2%, DCN: 29.1% and M/I: 1.81 in 2003, DCO: 3.8%, DCN: 6.9% and M/I: 2.38 in 2015).^14^, ^54^ This improvement in data quality standards was due to the selection of selected cancer‐designated hospitals in 2007 to promote cancer control programs by the Ministry of Health, Labour, and Welfare. Candidate hospitals to have high‐quality cancer registries were required,^55^, ^56^ which lead to an increase in the number of registrations and a dramatic decrease in the number of NOS cases, with detailed allocated to subsites. Therefore, the observed trends in incidence in our study might simply be an artifact representing an improvement in data quality.^14^ Accordingly, we cannot totally deny this possibility because data from three of the population‐based cancer registries with stable high quality during the observation period indicated a gradual upward trend in the HNC incidence in total and among subsites. However, consistent results in analyses limited to these three registries during the observation period might indicate that any bias due to the improvement in data quality would have had minimal impact on the observed trend in incidence. Trends for all HNC and subsites should be continuously monitored from 2016 using data from population‐based cancer registries which meet international data quality standards.

In conclusion, we identified trends in HNC incidence by subsite between 1993 and 2015 in a large representative Japanese population based on population‐based cancer registries. These trends might be due to changes in lifestyle factors in Japan. These results are crucial for the setting of public health priorities.

## ETHICS STATEMENT

This paper has not been published in this or a substantially similar form (in print or electronically, including on a web site), nor accepted for publication elsewhere, nor is it under consideration by another publisher. This study was performed in accordance with the Declaration of Helsinki.

## CONFLICT OF INTEREST

All authors declare no conflict of interest associated with this study.

## AUTHOR CONTRIBUTIONS

Daisuke Kawakita , Keitaro Matsuo, Hidemi Ito were involved in study design and data interpretation. All authors critically revised the report, commented on drafts of the manuscript, and approved the final report.

## Supporting information

Fig S1

Table S1

## Data Availability

The data that support the findings of this study are available on request from the corresponding author. The data are not publicly available due to privacy or ethical restrictions.
