# Supplementary figures and images for: Trends in the incidence of head and neck cancer by subsite between 1993 and 2015 in Japan
Source: Cancer Med. 2022 Jan 14;11(6):1553–60. doi: 10.1002/cam4.4539 (PMC8921930; doi:10.1002/cam4.4539)

**(a)****Lip cancer**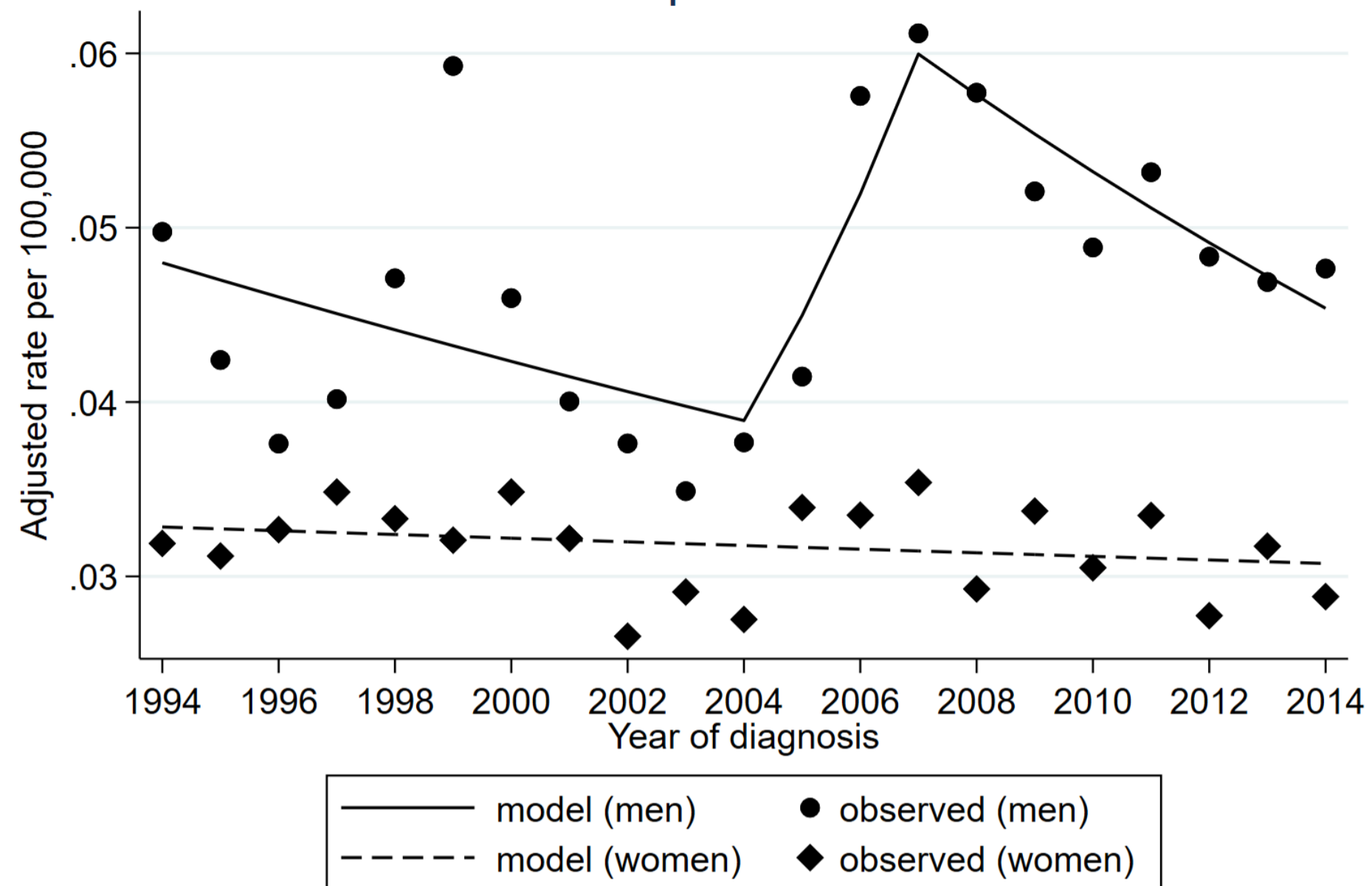**(b)****Middle ear cancer**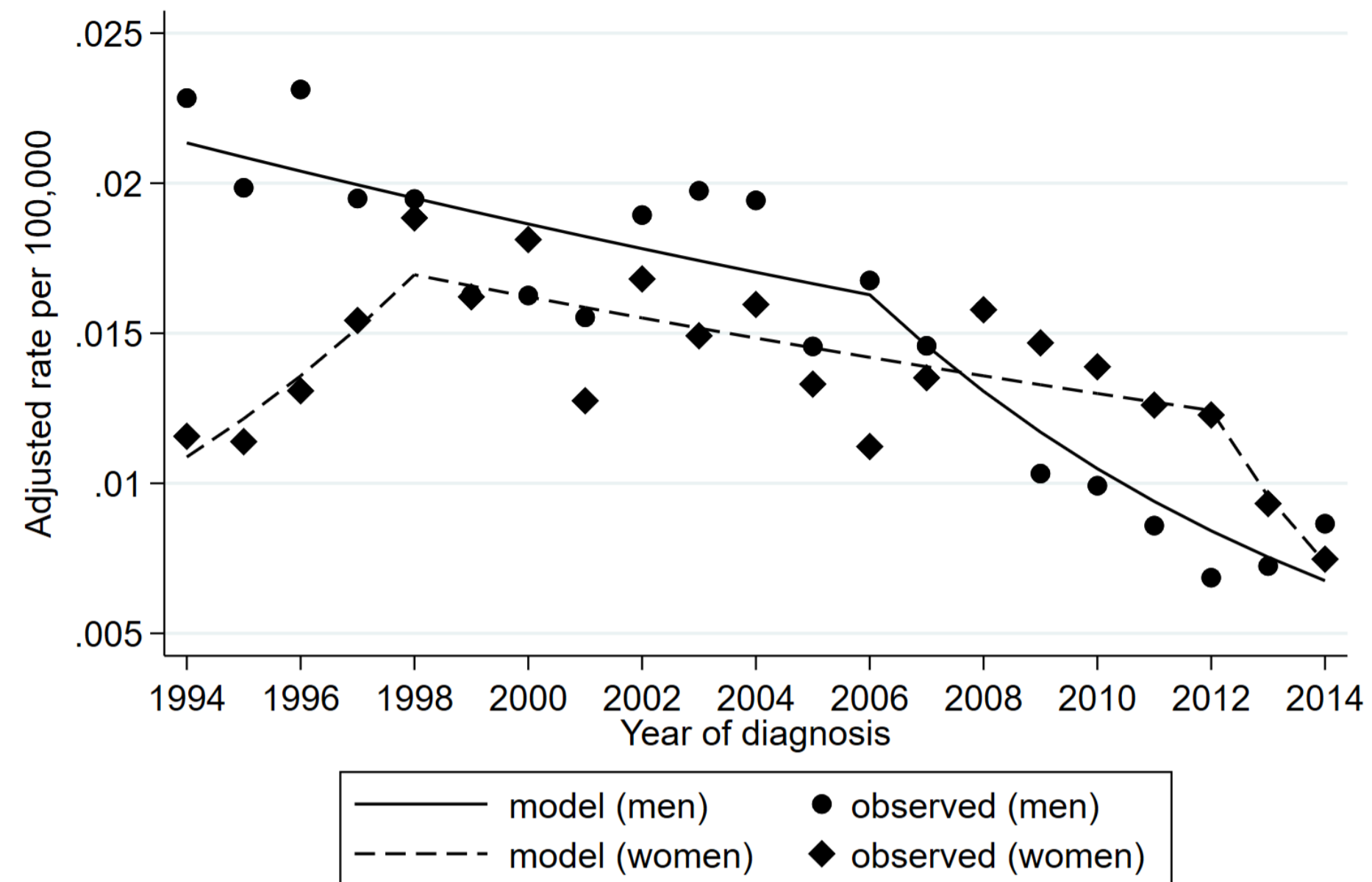**(c)****NOS cancer**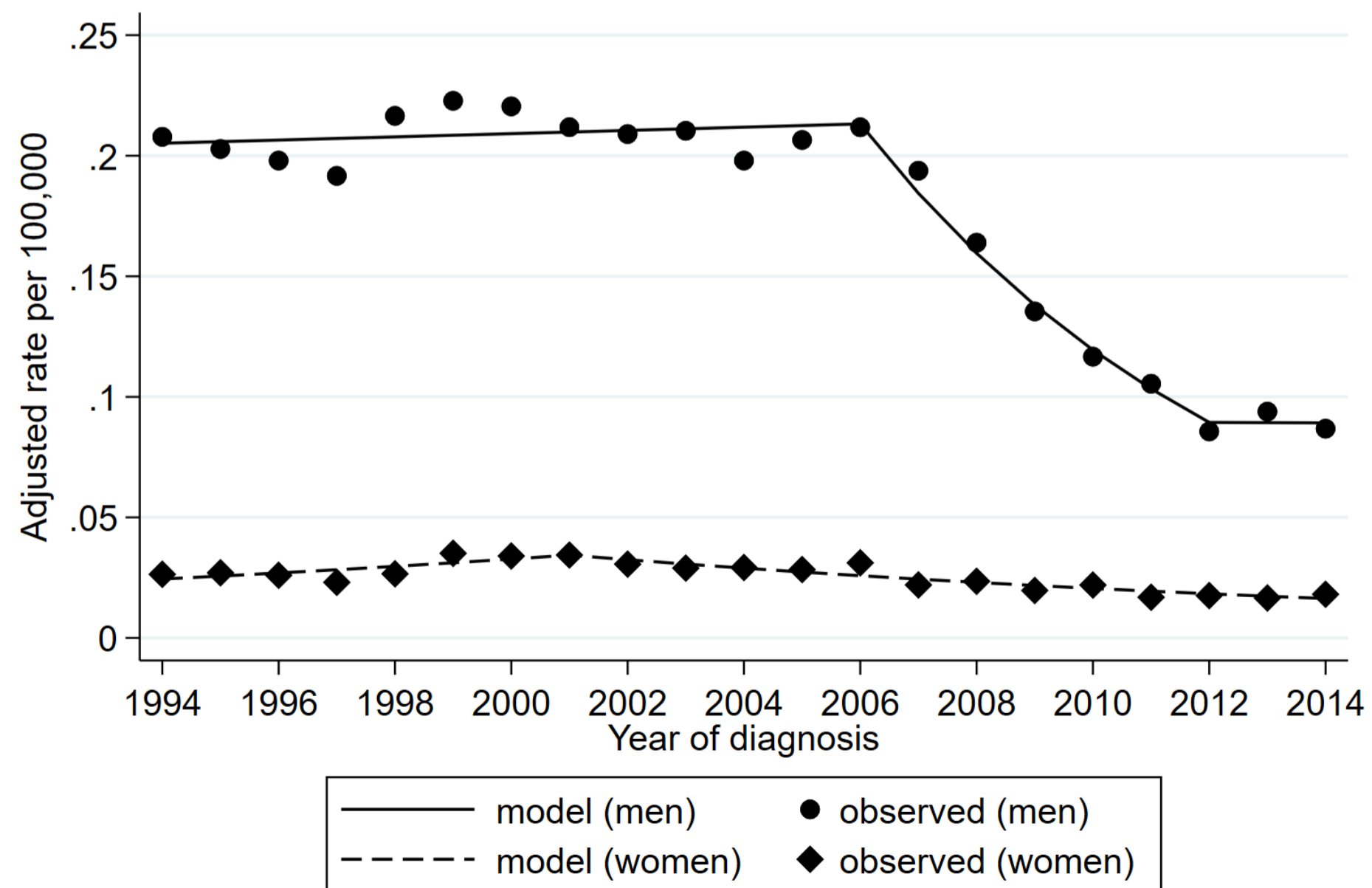

Supplement: Supplementary file 1 — Fig S1 [file CAM4-11-1553-s001.pdf]
