# Supplementary material for: Trends in the incidence of head and neck cancer by subsite between 1993 and 2015 in Japan
Source: Cancer Med. 2022 Jan 14;11(6):1553–60. doi: 10.1002/cam4.4539 (PMC8921930; doi:10.1002/cam4.4539)
Supplement: Supplementary file 2 [file CAM4-11-1553-s002.docx]

**Supplementary Table. Joinpoint analysis for Lip, Middle ear and NOS cancer between 1993 and 2015 using the moving average of incidence rate in 3-year category**

| Subsite | Men | | | Women | | |
|---|---|---|---|---|---|---|
| | Year | APC | 95% CI | Year | APC | 95% CI |
| Lip | 1994-2004 | -2.1 | -5.3, 1.2 | 1994-2014 | -0.3 | -1.0, 0.3 |
| | 2004-2007 | 15.5 | -21.0, 68.8 | | | |
| | 2007-2014 | -3.9 | -8.2, 0.6 | | | |
| Middle ear | 1994-2006 | -2.2* | -4.4, -0.1 | 1994-1998 | 11.7 | -1.2, 26.4 |
| | 2006-2014 | -10.4* | -14.7, -5.9 | 1998-2012 | -2.2* | -4.1, -0.3 |
| | | | | 2012-2014 | -23.1 | -49.9, 18.1 |
| NOS | 1994-2006 | 0.3 | -0.5, 1.1 | 1994-2001 | 5.0* | 0.8, 9.4 |
| | 2006-2012 | -13.5* | -16.1, -10.7 | 2001-2014 | -5.6* | -7.1, -4.0 |
| | 2012-2014 | -0.1 | -15.7, 18.4 | | | |

Abbreviations: APC, estimated annual percent change (age-standardized to the world population); CI, confidence interval; NOS, oral cavity or pharynx not otherwise specified.

* APC is statistically significantly different from zero (two-sided $p<0.05$).
